# Dataset on the solid-liquid separation of anaerobic digestate by means of wood ash-based treatment

**DOI:** 10.1016/j.dib.2022.108536

**Published:** 2022-08-12

**Authors:** Alejandro Moure Abelenda, Kirk T. Semple, George Aggidis, Farid Aiouache

**Affiliations:** a School of Engineering, Lancaster University, Lancaster LA1 4YW, UK; b Lancaster Environment Centre, Lancaster University, Lancaster LA1 4YQ, UK

**Keywords:** Acidification, Sorption, Phase fractionation, Mitigation of greenhouse gases emissions, Prevention of phosphate leaching, Circular economy, Bioenergy residues, pHzpc, pH of zero-point charge, PVWD, post-harvest vegetable waste digestate, WBA, wood bottom ash, WFA, wood fly ash, WI, water-insoluble, WS, water-soluble

## Abstract

Wood bottom and fly ashes were added to the anaerobic digestate using sulfuric, hydrochloric, nitric, and lactic acids, as pH conditioners and sorption activating agents. Minimum (pH of zero-point charge), mild, and severe acidification of the samples were tested. The solid-liquid separation achieved was accounted visually and with the measurement of the masses of water-soluble extract and water-insoluble material isolated. The average mass of the blend was 36.61 ± 0.68 g, including the extractant agent that was prepared with the commercial acids and ultrapure milli-Q^Ⓡ^ water. During the 144-h incubation of the mixtures at 20 °C and 0.17 x g, the shares of the solid and liquid were determined by centrifugation of the destructive samples at 3,130.40 x g for 5 minutes and 3-µm filtration of the supernatant. Before weighing the water-insoluble material, both the pellet that remained in the tube and the filter cake were dried at 70 °C until reaching constant weight. There was a significant increase in the amount of water-insoluble phase of the wood bottom ash due to the activation with lactic and sulfuric acids. The treatment of the wood fly ash and the agrowaste digestate with the hydrochloric acid showed an increase in the formation of water-soluble extract, in direct relation with the acidic pH of the blend. The characterization of the pH of the WS extract was performed with a Mettler Toledo^Ⓡ^ Seven Compact^TM^ S220 pH/Ion meter. The conditions of this process can be further optimized and each of the fractions can be characterized, in terms of nutrient content, to confirm the efficiency of the separation. More complex and ambitious processes can be designed for combining the wood ash and the anaerobic digestate. The performance of this treatment involving wood ashes and commercial acids can be extrapolated to other type of organic manures with a moisture content of approximately 95%, to improve their management in terms of reducing the cost of storage and transportation for land application below £5 per tonne.

## Graphical abstract

**Figure:**
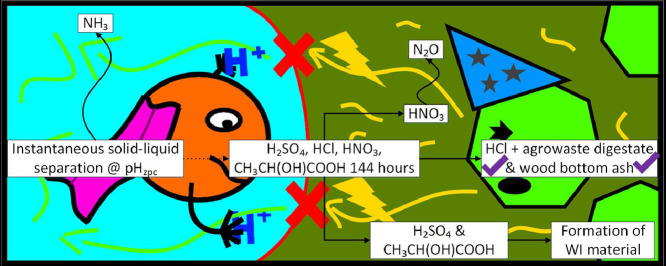

**Specifications Table**

**Table:** 

Subject	Renewable Energy, Sustainability and the Environment
Specific subject area	Stabilization of organic manures using additives to improve their properties as fertilizers and reduce the greenhouse gases emissions and excessive leaching of nutrients.
Type of data	Video Graphs
How the data were acquired	Video recording (Motorola moto g(6) play 13-megapixel rear camera with f/2 lens), scale balance (Waagenet Model AH-300V), and pH-meter (Mettler Toledo^Ⓡ^ Seven Compact^TM^ S220 pH/Ion meter). The fractionation of the samples of wood ash and anaerobic digestates under several treatments and blending conditions was done during their incubation in a closed chamber [1]. The effect of the blending the wood ash with the acidified anaerobic digestate was recorded. After the separation of the liquid extract and the solid fraction, their masses were accounted. Subsequently, the pH was measured in the fluid phases.
Data format	• Raw: Video recording of the finding in the laboratory upon blending the wood ash with the acidified anaerobic digestate. • Analyzed: The data were plotted in graphs and the trend of each treatment was differentiated from the others by applying the 2-way ANOVA test (p < 0.05). • Filtered: Removal of outlier value in the 4 replicates of each condition using the z-score method and the interquartile range.
Description of data collection	Blends of wood ash and anaerobic digestate were prepared using sulfuric, hydrochloric, nitric, and lactic acids to optimize the solid-liquid separation. The 144-h incubation of the blend was conducted at 20 °C and 0.17 x g. The impact of the strategy of preparation on the share of solid material and liquid extract in the blend was accounted by isolating these fractions in the destructive samples. The factors considering for designing the experiments were the level of acidification of the samples of wood ash and anaerobic digestate and the time of incubation.
Data source location	• Institution: Lancaster Environment Centre, Lancaster University • City/Town/Region: Lancaster, Lancashire • Country: United Kingdom
Data accessibility	Repository name: Lancaster University's Institutional Repository DOIs: https://doi.org/10.17635/lancaster/researchdata/531[2] https://doi.org/10.17635/lancaster/researchdata/550[3] https://doi.org/10.17635/lancaster/researchdata/549[4]
Related research article	Moure Abelenda A, Semple KT, Herbert BMJ, Aggidis G, Aiouache F. Valorization of agrowaste digestate *via* addition of wood ash, acidification, and nitrification. Environ Technol Innov 2022:102632. https://doi.org/10.1016/j.eti.2022.102632.

## Value of the Data

• These data highlight the potential of the wood ash-based treatment of the anaerobic digestates to achieve the solid-liquid separation, in addition to ensuring the chemical stabilization and an appropriate nutrient ratio. The solid-liquid separation of organic manures with a moisture content greater than 90 % is widely investigated through techniques of thermal drying, filtration, and flocculation [5].
• The use of organic and inorganic material as soil amendments and crop fertilizers is widely investigated. Particularly, the acidification has received plenty of attention for preserving the properties of the organic materials and improve the management of their nutrients. However, scarce evidence is reported on the capabilities of this technique to attain the solid-liquid separation of manures [6].
• The detailed procedure to obtain the solid and liquid phase separation is described in this manuscript, hence the conditions of this process can be further optimized and each of the fractions can be characterized, in terms of nutrient content, to confirm the efficiency of the separation. More complex and ambitious green processes can be designed for combining the wood ash and the anaerobic digestate.
• The data reported in this article could be generalized to other additives (aluminum sulfate, iron chloride, biochar, dolomite, zeolites and other sorptive materials) employed in the agroindustry to treat animal manure and slurry [7], [8], [9], [10].

## 1 Data Description

**Fig. 1:**
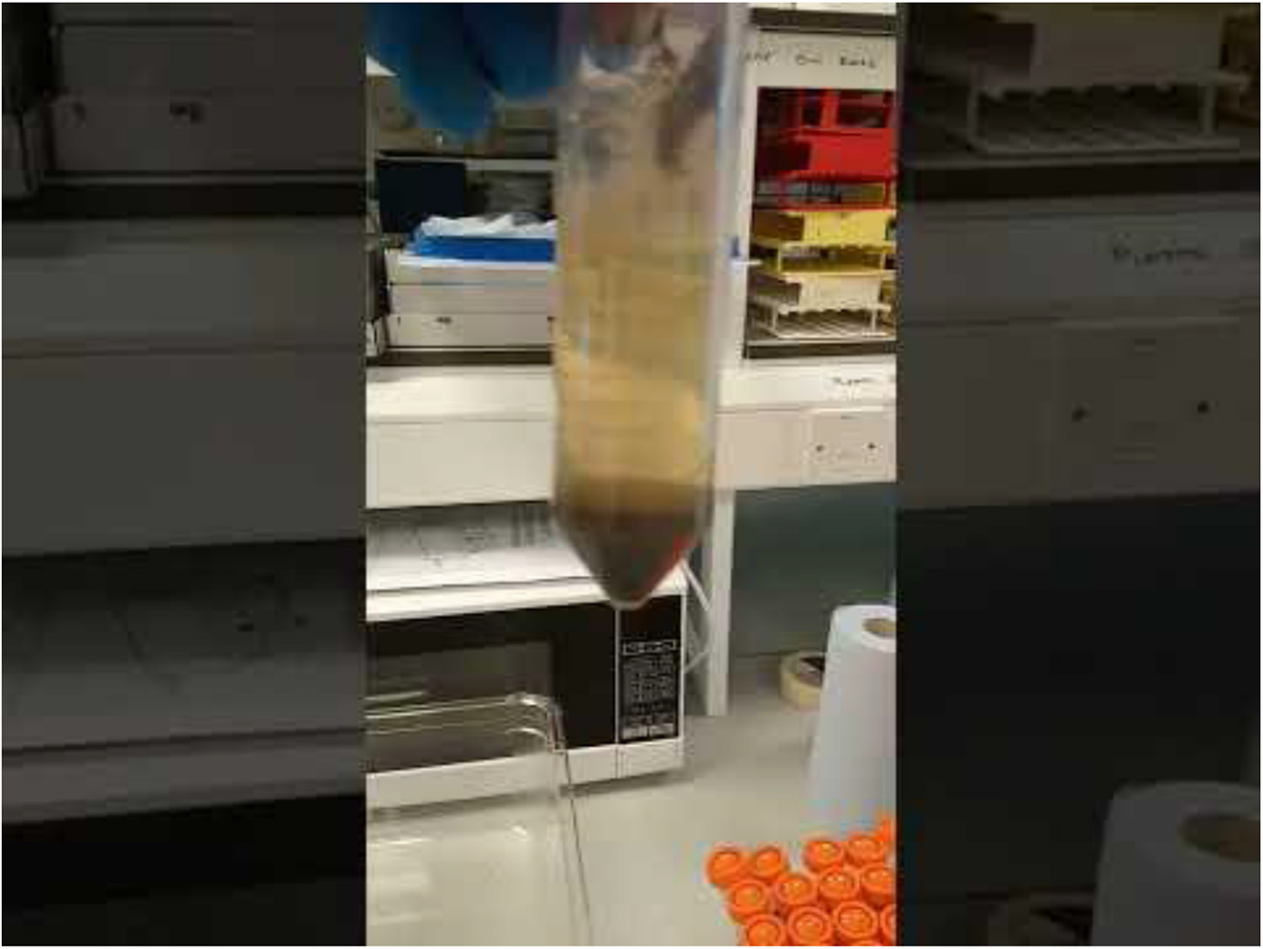
Thumbnail of the video shown the flocculation and settling of the anaerobic digestate by means of the wood ash based treatment as described in Moure Abelenda et al. [2].

The first raw data that is included in this article is a video (mp4 file; Fig. 1) that has been upload to the Lancaster University's Institutional Repository and the details can be found in the reference list [2]. The video shows how the flocculation and settling was achieved spontaneously at the pH of zero-point charge (pH_zpc_) of the wood ash [11], straight after blending the two waste streams (*i.e.*, the acidified anaerobic digestate and the wood ash), without the use of centrifugation. Further treatment of the liquor *via* aeration or addition of antifoam chemicals could remove the bubbles from the surface of the liquid.

**Fig. 2:**
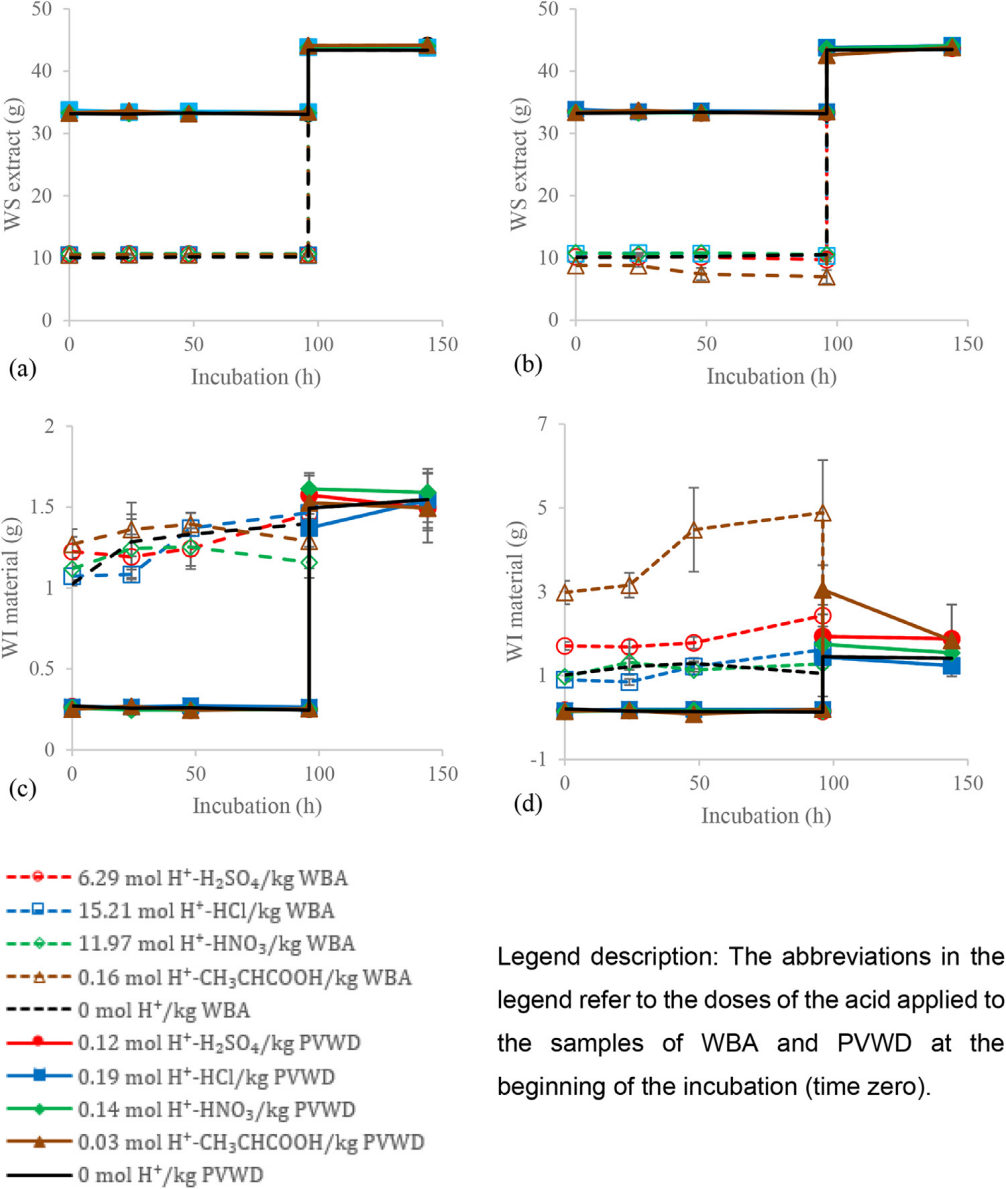
Initial and final fractionation of 1.28 ± 0.27 g of WBA and 3.40 ± 0.20 g of PVWD before and after blending (at the 96 hours) under 5 acidification conditions: (a) Initial WS extract, (b) final WS extract, (c) initial WI material, and (d) final WI material (n = 3). Reproduced with the permission of Journal of Environmental Chemical Engineering (Elsevier) [12].

However, the use of mild acidification was found not to complete mitigate the NH_3_ volatilization and for this purpose the severe acidification of the anaerobic digestate and the wood ash samples were tested [12]. According to Regueiro et al. [9], at ambient temperature (20 °C), a minimum incubation of 2 weeks is required to attain the stabilization. Fig. 2 corresponds to Figure S.6 of Moure Abelenda et al. [12]. Fractionation of the treated wood bottom ash (WBA) and agrowaste digestate (PVWD) with four different acids (*i.e.*, H_2_SO_4_, HCl, HNO_3_, and CH_3_CH(OH)COOH) before and after blending the samples at the 96 hours of incubation at 20 °C and 0.17 x g in 50-mL Corning^Ⓡ^ tubes. The initial amounts of water-insoluble (WI) material and water-soluble (WS) extract were determined considering a dry matter of 98.89 ± 0.58 % and 7.57 ± 0.32 % for the WBA and the PVWD, respectively. The raw data associated to Fig. 2 were upload to the Lancaster University's Institutional Repository in a xlsx file and further details can be found in the reference list [3]. The excel file also contains the ANOVA tests (p < 0.05) to confirm the significant differences between the trends observed. Especially important is the fact that the treatments with sulfuric acid and lactic acid increased the WI material and decreased the WS extract of the WBA significantly (p < 0.05).

**Fig. 3:**
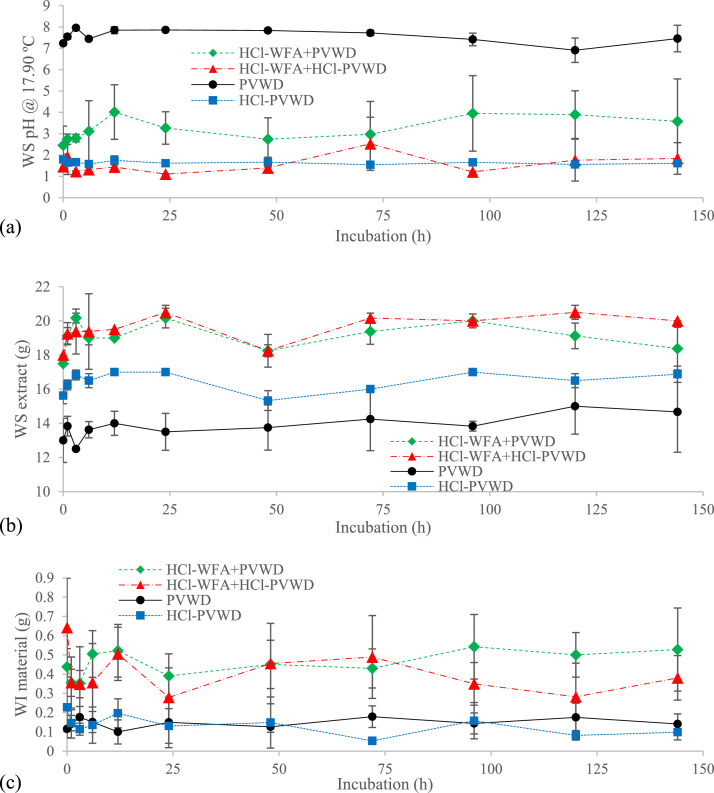
(a) pH of the 17.17 ± 2.56 mL WS extract and masses of the (b) WS and (c) WI fractions during the 144-h incubation of the destructive samples of HCl-WFA+PVWD, HCl-WFA+HCl-PVWD, PVWD, and HCl-PVWD (n = 4). Reproduced with the permission of Environmental Technology & Innovation (Elsevier) [1].

Because the best results were obtained with the HCl [12], this was the acid that was employed for the next investigation. It is important to highlight that the performance of the HNO_3_ in terms of promoting the solid-liquid separation and the dewatering of the samples was similar to the HCl, but the nitric acid affect the overall nitrogen balance and excess of NO_3_^−^ might promote N_2_O emissions upon land application [11]. Fig. 3 corresponds to Fig. S.2 and Fig. S.3 of Moure Abelenda et al. [1]. The raw data associated to Fig. 3 is available in an excel document that was uploaded to the Lancaster University's Institutional Repository [4]. The calculation of the outlier values with the interquartile range method is shown in red-shaded cells in the spreadsheet. The hydrolytic and dehydration effects of the acidification (Fig. 3a) could be seen in the mass of WS and WI fractions of the blend. In this way, the acidified digestate with hydrochloric acid (HCl-PVWD) had more WS fraction than the PVWD alone (Fig. 3b). Moreover, a lower the amount of WI material could be seen in the blend of wood fly ash (WFA) and PVWD severely acidified with hydrochloric acid (HCl-WFA+HCl-PVWD), compared to that of the mild acidification of the blend (HCl-WFA+PVWD) (Fig. 3c). It would be possible to calculate de separation capacity of each treatment as the ratio of WS extract to WI material. The results were 47.94 ± 17.26, 61.21 ± 35.03, 132.85 ± 103.63, and 178.33 ± 99.57 for HCl-WFA+PVWD, HCl-WFA+HCl-PVWD, PVWD, and HCl-PVWD (yellow-shaded cells in the spreadsheet). This would imply the hydrochloric acid acted as cationic surfactant, promoting the release of the bounded water, as described by Zheng et al. [5].

## 2 Experimental Design, Materials and Methods

The PVWD sample consisted of 5 kg of material, which was sourced at the end of the process line of the AD plant with a jerry can. Cooling down of the samples was allowed before placing them in suitable containers with ice for their transport by courier. Once received by the staff of Lancaster University, the samples were stored in a cold room (< 4 °C) until further use. These were considered “fresh” samples. Each ash sample (*i.e.*, WBA and WFA) received consisted of 10 kg of material, which was sent by the producer in a sealed plastic box to minimize the contact with the open atmosphere. Once at the university, each sample was milled to pass a 1 mm mesh, sieved, and placed in zip bags which were left under room temperature until further use. These were considered “fresh” samples. The complete characterization of the samples can be found at Moure Abelenda et al. [13]. Analytical grade sulfuric, hydrochloric, nitric, and lactic acids were employed for the acidification experiments.

The video was recorded with a Motorola moto g(6) play 13-megapixel rear camera with f/2 lens while performing the steps to attain the pH_zpc_ as previously reported [11]. The experimental unit that is examined in this video is similar to the one reported in the Fig. S.8 of [11]. The steps to reproduce these conditions of dewaterability of the anaerobic digestate with wood ash are:

1. Acidify the anaerobic digestate with a common dose (0.24 mol H^+^/kg anaerobic digestate) of a commercial acid (e.g., H_2_SO_4_, HCl, and HNO_3_): Add 10 mL of the acid solution to each gram of anaerobic digestate. In total, were around 20 mL of acid solution added to 2 g of anaerobic digestate. The CH_3_CH(OH)COOH is a weak acid and it would more convenient to compare its effect to that of the strong acids expressing the doses as equivalent mass of acid per kg of sample [11].
2. Add 10 mL of the deionized water to each gram of wood ash. In total, were around 5 mL of milli-Q^Ⓡ^ water added to 0.5 g of wood ash. The purpose of this step is to fluidize the wood ash for easy handling. It would be possible to apply several rounds of washing to the wood ash to remove the impurities and improve sorption capacity.
3. Blend the two fluidized waste streams: Flocculation and settling occurs spontaneously.

In the following investigation [12], in addition to use a commercial dose for treating the PVWD, the WBA was treated by means of severe acidification, as described in in the legend of Fig. 2. The extracting agents, which were prepared with an acid and ultrapure milli-Q^Ⓡ^ water, were added to the samples following a ratio of 10 mL for each gram of WBA or PVWD: 10.50 ± 0.22 g and 30.19 ± 0.20 g of extracting agents were added to 1.28 ± 0.15 g of WBA and the 3.40 ± 0.20 g PVWD, respectively.

Finally, the combinations of PVWD and WFA with the best acidification agent (*i.e.*, hydrochloric acid) were tested by Moure Abelenda et al. [1] (Fig. 3), with the doses 20 mol H^+^-HCl/kg WFA and 0.20 mol H^+^-HCl/kg PVWD. The isolation of the WS and WI fractions was done *via* 5-minute centrifugation at 3,130.40 x g, and subsequent 3-µm (Whatman No 44 filter paper) filtration of the supernatant. Before determining the weight of the total mass of WI material, both the pellet that remained in the 50-mL centrifuge Corning^Ⓡ^ tube and the filter cake were dried at 70 °C, until reaching constant weight measured with a Waagenet Model AH-300V scale balance. The characterization of the pH of the WS extract was carried out with a Mettler Toledo^Ⓡ^ Seven Compact^TM^ S220 pH/Ion meter.

## CRediT authorship contribution statement

**Alejandro Moure Abelenda:** Conceptualization, Data curation, Formal analysis, Investigation, Methodology, Validation, Visualization, Writing – original draft, Writing – review & editing. **Kirk T. Semple:** Conceptualization, Funding acquisition, Methodology, Project administration, Resources, Writing – review & editing. **George Aggidis:** Methodology, Project administration, Supervision, Writing – review & editing. **Farid Aiouache:** Conceptualization, Funding acquisition, Methodology, Project administration, Resources, Supervision, Visualization, Writing – review & editing.

## Declaration of Competing Interest

The authors declare that they have no known competing financial interests or personal relationships that could have appeared to influence the work reported in this paper.

## Data Availability

Fig. S.6 Impact of sulphuric, hydrochloric, nitric, and lactic acids in the preparation of a blend (Original data) (Lancaster University's Institutional Repository). Enhancement of the solid-liquid separation of the anaerobic digestate by means of acidification and subsequent addition of wood ash (Original data) (Lancaster University's Institutional Repository). Fig. S.2 & Fig. S.3 Valorization of agrowaste digestate via addition of wood ash, acidification, and nitrification (Original data) (Lancaster University's Institutional Repository).

## Appendix Supplementary materials

Image, application 1

Image, application 2

Image, video 3
